# Enhanced Collaborative Edge Intelligence for Explainable and Transferable Image Recognition in 6G-Aided IIoT

**DOI:** 10.3390/s25144365

**Published:** 2025-07-12

**Authors:** Chen Chen, Ze Sun, Jiale Zhang, Junwei Dong, Peng Zhang, Jie Guo

**Affiliations:** 1 Taihu Laboratory of Deepsea Technological Science, Wuxi 214082, China; zesun@hnu.edu.cn (Z.S.); zhangjiale@cssrc.com.cn (J.Z.); dongjunwei@cssrc.com.cn (J.D.); zhangpeng@cssrc.com.cn (P.Z.); 2 China Ship Scientific Research Center, Wuxi 214082, China; 3 The State Key Laboratory of Integrated Services Networks, Xidian University, Xi’an 710071, China; jguo@xidian.edu.cn

**Keywords:** image recognition, transfer learning, IIoT, edge intelligence, 6G

## Abstract

The Industrial Internet of Things (IIoT) has revolutionized industry through interconnected devices and intelligent applications. Leveraging the advancements in sixth-generation cellular networks (6G), the 6G-aided IIoT has demonstrated a superior performance across applications requiring low latency and high reliability, with image recognition being among the most pivotal. However, the existing algorithms often neglect the explainability of image recognition processes and fail to address the collaborative potential between edge computing servers. This paper proposes a novel method, IRCE (Intelligent Recognition with Collaborative Edges), designed to enhance the explainability and transferability in 6G-aided IIoT image recognition. By incorporating an explainable layer into the feature extraction network, IRCE provides visual prototypes that elucidate decision-making processes, fostering greater transparency and trust in the system. Furthermore, the integration of the local maximum mean discrepancy (LMMD) loss facilitates seamless transfer learning across geographically distributed edge servers, enabling effective domain adaptation and collaborative intelligence. IRCE leverages edge intelligence to optimize real-time performance while reducing computational costs and enhancing scalability. Extensive simulations demonstrate the superior accuracy, explainability, and adaptability of IRCE compared to those of the traditional methods. Moreover, its ability to operate efficiently in diverse environments highlights its potential for critical industrial applications such as smart manufacturing, remote diagnostics, and intelligent transportation systems. The proposed approach represents a significant step forward in achieving scalable, explainable, and transferable AI solutions for IIoT ecosystems.

## 1. Introduction

With the development of sensing, data analysis, and communication techniques, staggering numbers of devices have been connected to the Internet. It was previously reported that by 2021, the number of smart devices would reach 28 billion, among which the number of IoT devices would be approximately 15 billion [1,2,3,4,5,6,7]. In the last decade, industrial standards and infrastructures have substantially evolved, particularly due to the amalgamation of the IoT paradigm, referred to as the industrial IoT (IIoT). Innovation solutions for industries that save operational costs and enable high system reliability are maturing with the improvement of the IIoT [8,9,10].

In the era of sixth-generation cellular networks (6G), there will be a unifying platform for reaching the goal of the “Internet of Everything”. 6G will support various emerging applications and scenarios in the IIoT, e.g., long-range medical treatment, augmented reality (AR) and virtual reality (VR) scenarios [11], high-definition video surveillance, smart transportation [12], etc. Since the advantages of 6G include high-bandwidth, low-latency, large-scale, and low-cost networks, the 6G-aided IIoT has also become a hot topic in the academic and industrial research. Among the various applications of the 6G-aided IIoT, image recognition plays a significant role due to the wide deployment of image and video sensors. Excellent image recognition methods offer the ability to accurately identify people, vehicles, and other objects, which can be used to evaluate workers’ performance or monitor shipments of products in a processing line. Therefore, these methods will improve the production efficiency of the manufacturing industry and will benefit smart homes, long-range medical treatment, and so forth in the long run [12,13,14].

However, the image recognition algorithms employed in 6G-aided Industrial Internet of Things (IIoT) systems [15,16] demand an ultra-low latency and high reliability, making the traditional cloud-based processing methods inadequate. To address these limitations, a mobile edge computing (MEC) architecture has been introduced. By positioning the computing resources closer to end users, MEC significantly reduces service latency and enhances operational efficiency. Concurrently, the advancement of deep learning and artificial intelligence (AI) has given rise to a new paradigm known as edge intelligence, which integrates the computational advantages of MEC with the cognitive capabilities of AI [17,18,19]. For example, lightweight intelligent surveillance methods tailored to edge devices have been proposed to improve video monitoring performance; such approaches have demonstrated an enhanced detection accuracy by leveraging edge-based models [17]. A robust face recognition technique based on dynamic features has also been developed to withstand presentation attacks, ensuring higher trustworthiness in identity verification [20]. Additionally, a hybrid neural architecture combining convolutional, residual, and capsule networks was introduced to boost the facial expression recognition accuracy in IIoT contexts by integrating texture information into parallel pathways [21]. Further emphasizing the potential of this paradigm, research has shown that deploying AI models directly on edge devices offers a powerful solution to overcoming current challenges in computer vision tasks such as object recognition, image segmentation, and classification [22].

Although these image recognition methods based on edge intelligence have already been studied, the explainability of image recognition methods and the relevance between MEC servers have largely been ignored. In [23], the authors mentioned that the “collaborative edge” is one of the most promising applications, which can fuse geographically distributed data in terms of a virtual shared data view. However, currently, for image recognition, the intelligent model is more significant than the image data itself since if the image recognition model can be shared, the training process of the model on MEC servers can be largely simplified. Under this condition, “collaborative edge intelligence” is an essential approach to sharing intelligence. To address this issue, exploring explainable and transferable image recognition methods is essential. Once an image recognition model based on deep learning is established on one MEC server, it can also be applicable to other servers by introducing transfer learning methods.

It is true that prototype-based explanation and MMD domain adaptation have been studied separately. However, IRCE is the first framework (to our knowledge) that combines prototype-driven explainability with multi-edge domain adaptation in an IIoT/6G context. Existing interpretable models like ProtoPNet introduce a self-explaining prototype layer but assume a single (often static) domain. Likewise, many works on domain adaptation (e.g., MRAN) have extended MMD to various features but have not considered interpretability or an edge deployment scenario. IRCE bridges this gap: we embed a prototype layer (inspired by ProtoPNet) within a distributed edge network, and we use the local MMD loss to share knowledge across edges. For example, ProtoPNet shows how to incorporate a prototype layer for interpretability, but it does not address domain shifts or edge collaboration. On the other hand, MRAN introduces a multi-representation domain adaptation network that “extends Maximum Mean Discrepancy (MMD) to compute the adaptation loss”, yet MRAN uses only the global MMD on combined features and involves no mechanism for an explanation. Similarly, advanced domain adaptation models like DDASLA use the LMMD and attention to align the features, but they are focused on single-device inference and make no attempt to visualize or explain the decisions. In contrast, IRCE integrates these ideas: it uses the LMMD (similar in spirit to other subdomain alignment methods) specifically across edge servers in a collaborative IIoT environment, and it simultaneously provides pixel-level explanations via prototypical parts.

Since MEC servers are geographically distributed, the datasets and the environments of different MEC servers can be considered as different domains. Transfer learning, also known as domain adaptation, can be used to reduce the gap between the source domain and the target domain [24,25,26]. Traditional transfer learning methods for image recognition are based on global domain adaptation methods, which will result in low accuracy due to the deficiency of fine-grained local information. To address this issues, in this paper, we propose a novel image recognition method based on the 6G-aided IIoT, namely “IRCE”, which aims to overcome the disadvantages of the traditional time-consuming image recognition approaches based on deep learning. The main contributions of this paper are as follows.

(1) We propose an explainable feature extraction network instead of the traditional convolutional neural network. It has the ability to visualize the image features as prototypes which can easily be understood. Therefore, the proposed method can provide traceable reasons during identification.
(2) We present a deep subdomain adaptation transferable recognition method based on the MEC architecture, which is a significant application scenario in the 6G-aided IIoT. The domain adaption method employs the local maximum mean discrepancy (LMMD) to capture the fine-grained information for recognition. Even though the model is transferred to another edge server, the recognition accuracy is preserved.
(3) We design an end-to-end training method to optimize the parameters of different layers and the loss function of the proposed IRCE network, which contains the LMMD loss, the cross-entropy loss, the cluster loss, and the separation loss to ensure image recognition accuracy.

The remainder of this paper is organized as follows. Section 2 introduces the background knowledge on the proposed method, including the MEC architecture, image recognition based on deep learning, and the transfer learning module between different MEC servers. Section 3 provides the architecture of the proposed IRCE. The simulation results are given in Section 4, followed by the conclusions in Section 5.

## 2. Background

In this section, the background knowledge on the proposed IRCE method is given, including the MEC architecture, image recognition, and transfer learning.

### 2.1. The MEC Architecture

In a traditional cloud computing structure, end users send requests to the cloud, and then the cloud sends the results to users. Although the computing and storage abilities of the cloud are powerful, the delay in its services may be quite significant. In the MEC architecture [23,27], edge servers are deployed along the path between users and the cloud. Edge computing refers to the enabling technologies allowing computation to be performed at the edge of the network. Offloading, caching, data storage, and processing can be performed on an edge server.

**Figure 1:**
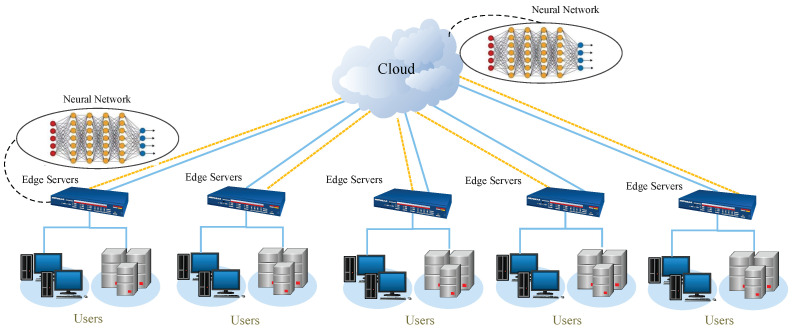
Edge intelligence based on mobile edge computing (MEC) framework.

As shown in Figure 1, the MEC architecture mainly consists of three parts, namely the users’ plane, the edge plane, and the cloud plane. Since the cloud has a more powerful computation capability, in the MEC architecture, the training process is performed on the cloud, and the generated model is then transferred to other servers. The generated model is transmitted between the cloud and the edge server. Compared with the MEC architecture, traditional centralized cloud computing often has a higher latency, which is not suitable in 6G scenarios. The advantages of MEC mainly include low latency, mobile energy savings, and privacy/security enhancements. MEC is one of the most important technologies for the next-generation Internet. Combining the MEC architecture with neural networks, edge intelligence emerges and has a variety of applications [17,18,19].

### 2.2. Image Recognition Based on Deep Learning

Image recognition methods related to the IIoT can be classified into many categories according to the objects that need to be recognized: for example, traffic sign recognition [28], text recognition [29], remote sensing image recognition [30], face recognition [31], etc. Although the emphases of these studies are different, their image recognition processes mainly include image segmentation, feature extraction, and classification [32]. Recently, the commonly used image recognition methods have mainly included kernel methods [33] and deep learning (DL) methods [34]. Kernel methods map the original features to a higher-dimensional space and introduce nonlinearity into the decision function, while image recognition algorithms based on deep neural networks refer to the use of large-scale and deep neural networks to learn the extracted features based on a large number of datasets, generalize the real scene, and complete the classification of the image. With the development of artificial intelligence, image recognition methods based on DL have achieved an outstanding performance in terms of both their accuracy and efficiency. However, due to the ultra-high-dimensional nonlinearity of deep-learning-based models, the explainability of these approaches is usually not favorable.

In some real-world IIoT applications, the internal causes that trigger the image recognition result may be more valuable than the recognition result itself, especially in certain medical image analysis and smart transportation scenarios [35]. Therefore, it is significant to explore explainable image recognition methods for judging the credibility of the decisions made by a trained recognition model.

### 2.3. Transfer Learning

Although image recognition methods have witnessed significant improvements, most of these methods are based on the common assumption that the training and testing data belong to the same feature space. However, in real scenarios, abundant training data that pertain to the same space or the same distribution as the testing data may not always be available. Therefore, transfer learning, which has the ability to transfer the learned knowledge to more applications, has emerged.

**Figure 2:**
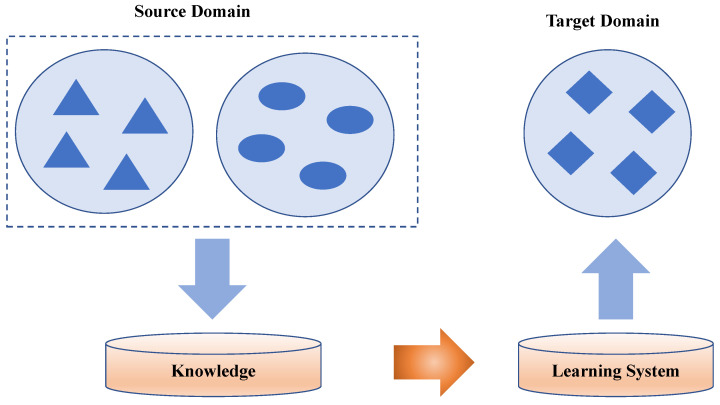
Illustration of transfer learning.

As shown in Figure 2, the shape in the circle denotes the category of the data distributions. The distributions of the source domain and target domain data are different. The learned knowledge already exists in the source domain. The learning system uses methods to try to explore the inner correlations between the source domain and the target domain and then transfer the acquired knowledge into the target domain. Transfer learning can be considered a special learning paradigm wherein part of/all of the training data used are under a different distribution than that of the testing data [24,25,36]. Meanwhile, to measure the similarity of the target and source domains, the most commonly used criterion is the maximum mean discrepancy (MMD), namely

(1) $$Dist_{k}\left(D^{s},D^{t}\right)={∥\frac{1}{n_{s}}\sum_{i=1}^{n_{s}}\phi \left(x_{i}^{s}\right)-\frac{1}{n_{t}}\sum_{i=1}^{n_{t}}\phi \left(x_{i}^{t}\right)∥}^{2},$$

where $D^{s}$ and $D^{t}$ denote the data in the source domain and the target domain, respectively. $x_{i}$ is the *i*th feature vector, and ϕ(.) stands for the feature space mapping function.

Since MEC servers are geographically distributed, once a model is established on one server, it may not be applicable to other servers. In terms of this issue, transfer learning has become a powerful and essential technique for solving the domain adaptation problem with trained image recognition models based on DL.

## 3. The Proposed Scheme

**Figure 3:**
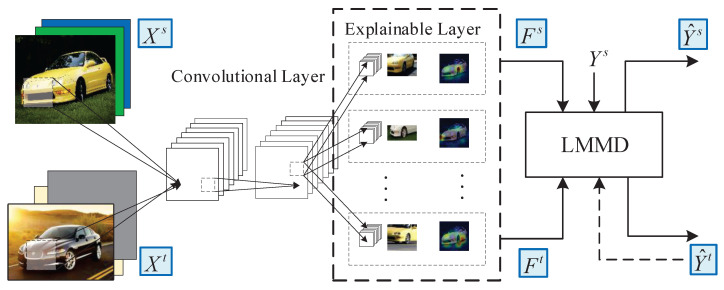
The proposed IRCE method.

In this section, the image recognition method “IRCE” is presented. As shown in Figure 3, we use the convolutional layer of the traditional convolutional neural network to extract the visual features of images in the source domain and the target domain, which are $X^{s}$ and $X^{t}$, respectively. IRCE is built on a ResNet backbone, which consists of 50 convolutional layers organized into bottleneck residual blocks, as shown below. Each “bottleneck” block uses three convolutions (1 × 1, 3 × 3, 1 × 1) with a skip (identity) connection. This design enables very deep feature extraction without vanishing gradients. IRCE uses this as the feature backbone (purple), with an added prototype layer and the loss components explained below. The explainable layer is then used to generate the prototypes that can represent the visualized characteristics of images. Finally, the output features of the source domain $F^{s}$, the target domain image features $F^{t}$, the real tag of an image in the source domain ${\hat{Y}}^{s}$, and the tag obtained for a target domain image through the network ${\hat{Y}}^{t}$ are combined to calculate the LMMD value, which is then added into the total loss function for iterative updating. In this way, the proposed IRCE method is adapted to the target domain and can then be applied to different image recognition datasets. The main parts of the proposed method include the prototype visualization module, the analysis and reasoning module, and the sdeep subdomain adaptation module.

### 3.1. The Prototype Visualization Module

In this part, our purpose is to acquire the visual prototypes which correspond to the indispensable part of the explainable analysis and reasoning in Section 3.2.

To extract meaningful features from images, the convolutional layer of a convolutional neural network (CNN) is utilized as the first part of our image recognition network. For an image, pixel values can be regarded as two-dimensional matrices which are the input to the convolution layer. Then, the explainable layer follows. The purpose of the explainable layer is to use the information obtained from the training image to generate prototypes that can represent the main characteristics of the image.

After the final convolutional layer, IRCE inserts an explainable prototype layer similar to ProtoPNet. In this layer, for each class k, we learn a set of prototype vectors $p_{k,1},\ldots ,p_{k,M}$ in the latent feature space. During inference, the similarity between each prototype $p_{k,l}$ and patches of the ResNet feature map is computed to produce a prototype activation map. These activations are pooled (typically via global max pooling) to yield a score for each prototype, and a linear layer then combines the prototype scores into class logits. In effect, each prototype acts like a learned “part” of the class, and the network’s decision is based on how closely parts of the input match these learned parts. Because IRCE explicitly ties each prototype to a visual concept, the model’s reasoning can be traced: each prototype corresponds to a specific image region that the model deems representative of a class. To make the prototypes human-interpretable, we employ a prototype projection (push) step during training. Periodically, each prototype vector $p_{j}$ is “pushed” onto the nearest patch from a training image of the same class.

Prototype visualization refers to using the analysis and processing functions of the computer to show prototypes that people can easily recognize and understand. To obtain an explainable image recognition network, we need to visualize the prototypes. The visualization process is achieved by projecting the prototype onto the training image patches that belong to the same category as the prototype. For a prototype $p_{j}$, the iteration process can be formulated as

(2) $$p_{j}\leftarrow \arg\min_{z}{∥z-p_{j}∥}_{2},\;\;\;\tilde{z}\in \left\{\;\tilde{z}:\;\tilde{z}\in f\left(x_{i}\right)\right\},$$

where *z* represents the convolution output f(x), and $\tilde{z}$ represents the training image patch that is the closest to the prototype. The prototype $p_{l}^{k}$ is expressed as $q_{l}^{k}$ before projection and $h_{k}^{l}$ after projection; then, the nearest patch to $p_{l}^{k}$ in the training image after projection is

(3) $$z_{l}^{k}=\arg\min_{\tilde{z}\in f\left(x\right)}{∥\tilde{z}-h_{l}^{k}∥}_{2}.$$

Assume that a δ value exists that satisfies 0<δ<1, and $\theta =\min(\sqrt{1+\delta }-1,1-\frac{1}{\sqrt{2-\delta }})$. If the classification result is incorrect, then

(4) $${∥h_{k}^{l}-q_{k}^{l}∥}_{2}\leq \theta {∥z_{k}^{l}-q_{k}^{l}∥}_{2}-\sqrt{\varepsilon }.$$

If the classification result is correct, we have

(5) $$\begin{array}{c} {∥h_{k}^{l}-q_{k}^{l}∥}_{2}\leq \left(\sqrt{1+\delta }-1\right){∥z_{k}^{l}-q_{k}^{l}∥}_{2}\\ {∥z_{k}^{l}-q_{k}^{l}∥}_{2}\leq \sqrt{1+\delta }. \end{array}$$

The results show that after projection, in the worst case, the output logit of incorrect classification will mostly increase by $\Delta_{\max}=a\log\left(\left(1+\delta \right)\left(2-\delta \right)\right)$, where *a* represents the number of prototypes of each class. When the classification result is correct, the output logit will mostly decrease by $\Delta_{\max}$. Therefore, as long as the output logit between the two classes has a gap of more than $2\Delta_{\max}$, the projection will not affect the image classification result. This projection ensures that each prototype $p_{j}$ becomes exactly equal to some real training patch’s feature; it can then be visualized by mapping that feature back to the original image patch. This “looks like” mechanism is what makes the layer explainable: each prototype can be displayed as a small image patch that it has learned, as shown in ProtoPNet.

The image area corresponding to the prototype is the highly activated area, so this area can be used for visualization. First, using the activation map generated by the explainable layer, we can find the most activated part of the image. Then, the strongest activated part surrounds the smallest rectangular area. This rectangular area can represent the prototype after visualization.

### 3.2. The Explainable Analysis and Reasoning Module

**Figure 4:**
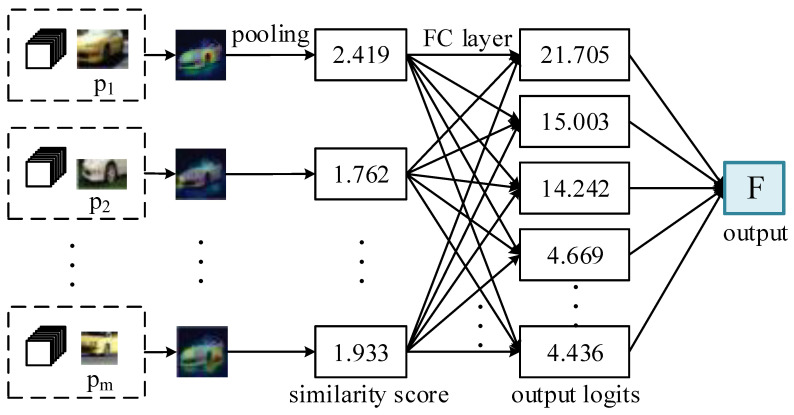
An illustration of the explainable layer.

The analysis and reasoning process of image recognition is mainly realized through the explainable layer; a detailed illustration of the explainable layer is shown in Figure 4.

When using the prototype generated by the explainable layer for classification, the distance calculated using the $L_{2}$ norm also represents the similarity score. According to the similarity score, we can obtain an activation map and generate a heat map which can clearly represent the similarity between the prototype and the image area. Then, we can obtain the class of the test image according to the class of the prototypes and obtain the final classification of the image according to the weighted sum of the similarity scores.

Mathematically, the classification of the network can also be expressed in terms of probability. We can explain it through Bayes Theorem:

(6) $$P\left(Y=k\left|X=x\right.\right)=\frac{P\left(X=x\left|Y=k\right.\right)P\left(Y=k\right)}{\sum_{j=1}^{K}P\left(X=x\left|Y=j\right.\right)P\left(Y=j\right)},$$

where $k\in \left\{1,...,K\right\}$. We use the latent space where the prototype is located to calculate the class conditional probability better. At the same time, the probability of X=x is the same for each class, which has the preconditions of the known category and the known image patches closest to the prototype. We then have

(7) $$\begin{array}{c} P(Y=k\left|X=x\right.)\\ =\frac{\Pi_{l}^{n_{k}}P\left(f_{l}^{k}\left(X\right)=f_{l}^{k}\left(x\right)\left|Y=k\right.\right)P\left(Y=k\right)}{\sum_{j=1}^{K}\Pi_{l}^{n_{k}}P\left(f_{l}^{j}\left(X\right)=f_{l}^{j}\left(x\right)\left|Y=j\right.\right)P\left(Y=j\right)}, \end{array}$$

where $f_{l}^{k}\left(x\right)=\arg\min_{z\in f(x)}{∥z-p_{l}^{k}∥}_{2}$. This represents the training image patch that is closest to the prototype.

### 3.3. The Deep Subdomain Adaptation Module

To improve the adaptation of the proposed image recognition method, transfer learning algorithms should be considered. In transfer learning algorithms, the MMD has been widely used to measure the discrepancy of the data distributions between the source domain and the target domain. Inspired by the LMMD method proposed in [24], we use the LMMD to align the distributions of the relevant domains.

(8) $$d_{H}\left(p,q\right)\overset{\Delta }{\;=}E_{c}{∥E_{p(c)}\left[\phi (X^{s})\right]-E_{q(c)}\left[\phi (X^{t})\right]∥}_{H}^{2},$$

where $X^{s}$ and $X^{t}$ are in the source domain and the target domain, respectively, and p(c) and q(c) are the distributions of the source domain and the target domain, respectively. The LMMD loss concentrates on the difference in the data distributions. By minimizing Equation (8), we can obtain the distribution for each subdomain in the same category.

We assume that the weight of each sample belonging to each category is $w_{c}$, and we can then rewrite Equation (8) as

(9) $${\hat{d}}_{H}\left(p,q\right)=\frac{1}{C}\sum_{c=1}^{C}{∥\sum_{x_{i}^{s}\in D_{s}}w_{i}^{sc}\phi \left(x_{i}^{s}\right)-\sum_{x_{j}^{t}\in D_{t}}w_{j}^{tc}\phi \left(x_{j}^{t}\right)∥}_{H}^{2}.$$

Given a source domain with $n_{s}$ labeled instances and a target domain with $n_{t}$ unlabeled points from different data distributions *p* and *q*, the deep networks will generate activations in layers. We can thus have

(10) $$\begin{array}{cc} {\hat{d}}_{l}\left(p,q\right) & =\frac{1}{C}\sum_{c=1}^{C}\left[\sum_{i=1}^{n_{s}}\sum_{j=1}^{n_{s}}w_{i}^{sc}w_{j}^{sc}k\left(z_{i}^{sl},z_{j}^{sl}\right)\right.\\ \; & +\sum_{i=1}^{n_{t}}\sum_{j=1}^{n_{t}}w_{i}^{tc}w_{j}^{tc}k\left(z_{i}^{tl},z_{j}^{tl}\right)\\ \; & \left.-2\sum_{i=1}^{n_{s}}\sum_{j=1}^{n_{t}}w_{i}^{sc}w_{j}^{tc}k\left(z_{i}^{sl},z_{j}^{tl}\right)\right]. \end{array}$$

### 3.4. The Loss Function

The learning objective of the proposed IRCE model is to optimize the performance and transferability of image recognition simultaneously. Specifically, the loss function of the proposed method $L_{total}$ includes the cross-entropy loss (CrsEnt), the cluster cost (Clst), the separation cost (Sep), and the LMMD loss ($d_{l}$), which can be calculated as follows.

(11) $$\begin{array}{c} \;L_{total}\;=\min_{P,w_{conv}}\frac{1}{n}\sum_{i=1}^{n}CrsEnt(h\circ g_{p}\circ f\left(x_{i}\right),y_{i})\\ \;\;\;\;\;\;\;\;\;\;\;\;\;\;\;\;\;\;\;\;\;\;\;+\lambda_{1}Clst+\lambda_{2}Sep+\lambda_{3}\sum_{l\in L}{\hat{d}}_{l}\left(p,q\right), \end{array}$$

where Clst, Sep, and $d_{l}$ are defined by

(12) $$\begin{array}{c} \begin{array}{cc} \;Clst\; & =\frac{1}{n}\sum_{i=1}^{n}\min_{j:p_{j}\in P_{y_{i}}z\in \mathrm{patches}\left(f\left(x_{i}\right)\right)}{∥z-p_{j}∥}_{2}^{2},\\ \;Sep\; & =-\frac{1}{n}\sum_{i=1}^{n}\min_{j:p_{j}\notin P_{y_{i}}z\in \mathrm{patches}\left(f\left(x_{i}\right)\right)}{∥z-p_{j}∥}_{2}^{2}. \end{array} \end{array}$$

The cross-entropy loss penalizes misclassification of the training data. Minimization of the cluster cost encourages each training image to have some latent patch that is close to at least one prototype of its own class. Minimization of the separation cost encourages every latent patch of a training image to stay away from prototypes which are not from its own class. Minimization of the LMMD minimizes the differences in the distributions between the two different datasets, namely the source domain and the target domain.

The loss function of IRCE combines several terms to jointly train feature extraction, prototype learning, and domain alignment. We use a standard cross-entropy classification loss $L_{\mathrm{CrsEnt}}$ on the final logits. In addition, we include prototype clustering and separation losses, as in ProtoPNet. Concretely, let f(x) be the feature map of input *x*, and let $p_{k,l}$ denote the *l*-th prototype for class *k*. The cluster* loss $L_{\mathrm{Clst}}$ encourages each training image to have *at least one* patch close to a prototype of its own class. Formally, for each image $x_{i}$ with a label $y_{i}$, we find the nearest feature patch $z\in f(x_{i})$ to any prototype of the class $y_{i}$ and penalize $∥z-p_{y_{i},j}{∥}^{2}$. The *separation* loss $L_{\mathrm{Sep}}$ encourages all latent patches of $x_{i}$ to stay far from prototypes from *other* classes. That is, for all prototypes $p_{k,j}$ with $k\neq y_{i}$, we penalize $\min_{z\in f(x_{i})}{∥z-p_{k,j}∥}^{2}$. These terms shape the latent space so that patches cluster around prototypes of their true class and are repelled from prototypes of other classes. IRCE adds a LMMD term $L_{\mathrm{LMMD}}$ to align the feature distributions across edge servers (domains). LMMD is an extension of the classic MMD that matches local class-conditional distributions. Roughly, for each class or subdomain, the LMMD minimizes the squared difference between the mean feature vectors in the source and target domains. In our implementation, we apply the LMMD between every pair of edge domains to encourage their features for the same class to coincide.

Prototype projection, though conceptually different, maintains a computational footprint comparable to that of a standard convolutional operation followed by global average pooling—a widely adopted mechanism in convolutional neural networks like ResNet. This similarity arises from the nature of the operations: both evaluate over all spatial patches of a given size, whether through minimum-distance matching (in our method) or average response accumulation (in traditional CNNs). As a result, integrating prototype projection into our architecture does not introduce an additional computational overhead during training. The dominant source of complexity remains in the image feature extraction module. Notably, our model, built upon a ResNet-34 backbone, is computationally more efficient than architectures like VGG-19. With approximately 3.6 billion floating-point operations (FLOPs), our network operates at roughly 18% of VGG-19’s 19.6 billion FLOPs. This efficiency ensures that the model remains lightweight and fully compatible with edge deployment requirements, such as on the Huawei Ascend 310 processor, which offers a peak capacity of 22T FLOPs.
**Algorithm 1:** Learning for the IRCE model. 
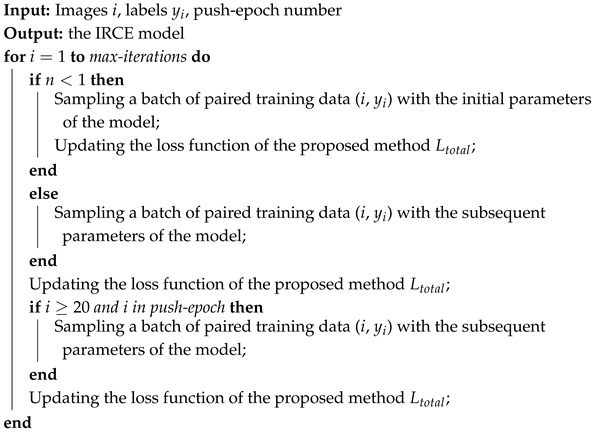


## 4. Simulation Results and Discussions

In this section, to prove the efficiency of the proposed IRCE method, four datasets, Office-31, Office-Home, ImageCLEF-DA, and Office-Caltech-10, are used. We have conducted several experiments by using IRCE for object recognition and comparing the results with those for other image recognition methods. Our algorithm 1 is implemented in Torch-1.1.0 with a Python 3.7 wrapper and runs on an Intel Core i7-9700k CPU with the NVIDIA GeForce RTX 2070 super-GPU.

### 4.1. The Datasets and Presetting

Office-31 is a commonly used dataset for domain adaptation, comprising 4110 images from 31 classes from three different data sources (domains) [37]: Amazon (A), which consists of images downloaded from the Internet, and DSLR (D) and Webcam (W), which contain images from digital cameras with different photographic criteria. To obtain sufficient evaluation results, we evaluate the object recognition algorithms with respect to all six transfer tasks: A→W, D→W, W→D, A→D, D→A, and W→A (the character “→” refers to the image recognition network transferring from the source domain to the target domain).

Office-Home is a recently released benchmark dataset for domain adaptation [38]. It contains 15,588 images from four different data domains: artistic images (A), clip art (C), product images (P), and real-world images (R). For each domain, the dataset includes 65 object categories collected in office and home scenarios. Similarly, the different object recognition algorithms are implemented in several transfer tasks: A→C, C→R, R→P, and P→A.

ImageCLEF-DA is a classical dataset from the domain adaptation challenge of ImageCLEF 2014 which contains the 12 common categories shared by the following three public datasets, each of which is considered a domain: Pascal VOC 2012 (P), Caltech-256 (C), and ImageNet ILSVRC 2012 (I). There are 50 images in each category and 600 images in each domain. We use all domain combinations and build six transfer tasks: I→P, P→I, I→C, C→I, C→P, and P→C.

Office-Caltech-10 is the most popular dataset for domain adaptation. There are four domains: C (Caltech), A (Amazon), W (Webcam), and D (DSLR). In fact, this dataset is constructed from two datasets: Office-31 (which contains 31 classes of A, W, and D) and Caltech-256 (which contains 256 classes of C).

For all of the transfer tasks, we use the Adam optimizer, where the batch size for training and testing is 64 and the model’s initial learning rate is 0.0001. In the first 20 epochs, the goal of the model is to learn the explainable part; therefore, we only optimize the convolutional layer parameters and the prototype layer parameters. The initial learning rate is set to 0.0001. In all subsequent epochs, we optimize the parameters of the ResNet layers, the convolutional layers, and the prototype layers. Their initial learning rate is set to 0.003. When the preset iteration times are achieved, we perform convex optimization to adjust the parameters of the last fully connected layer (the learning rate here is set to 0.0001). In the explainable part, we set the two respective hyperparameters of the cluster cost and the separation cost to 0.8 and −0.08. In the transfer part, we fix the adaptation factor lambda for the LMMD loss to suppress badness activations during the initial stages of training. We gradually change this from 0 to 1 using a progressive schedule: $\lambda_{\theta }=\frac{2}{\exp(\gamma^{\theta })}-1$ and γ=10 are fixed throughout the whole experiment.

### 4.2. Results and Discussion

In this section, to prove the explainability and transferability of the proposed method, the experiments are divided into three parts. In the first part, the structure of the latent space learned by the proposed IRCE is revealed. The experiment shows the closest local characteristic to a prototype in the images during the training and testing process. In addition, the reasoning process for the proposed image recognition method is visualized. The results show that after testing, the single image on the transferred model is also interpretable to some extent. The second part provides ablation studies to show the effectiveness of the different modules in the proposed method.

In the third part, we compare the transferability of the proposed IRCE with that of ResNet (the baseline method which refers to the structure of the proposed IRCE network without the explainable layer) and ProtoPNet (ResNet is a residual network that solves the problems of gradient explosion and gradient disappearance caused by the deepening of the network; ProtoPNet shows that the reasoning function in this network is demonstrated by the visual point of view). The average accuracy is used as the criterion to evaluate the model efficiency.

#### 4.2.1. Quantization of Results for Explainability

**Figure 5:**
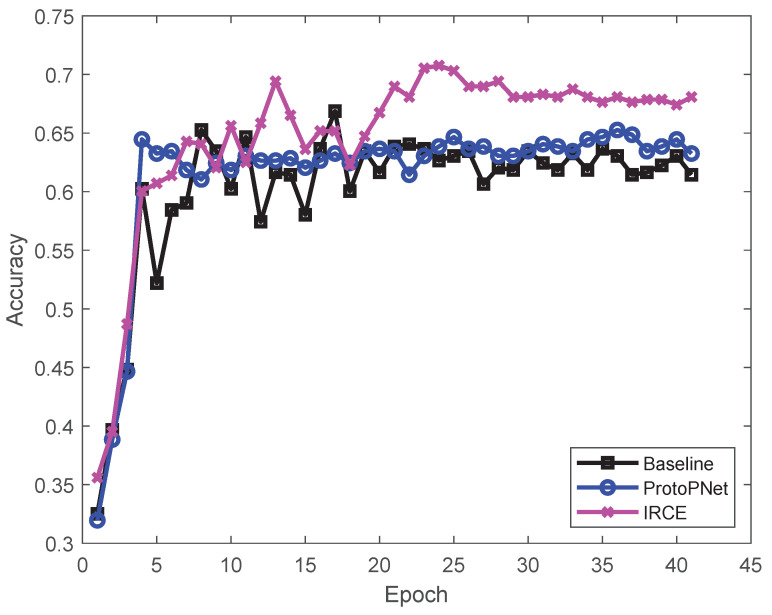
Comparison of image recognition accuracy with different numbers of epochs.

The classification accuracy of our IRCE (with various base ResNet 34) method for Office-31 and Office-Home is compared to that for the corresponding baseline model at the top of Table 1, Table 2 and Table 3: the first number in each cell gives the mean accuracy over three runs. To ensure the fairness of the comparison, the ResNet model and the ProtoPNnet model were trained on the same augmented Office-31 dataset as the corresponding IRCE. As shown in Figure 5 and the above two tables, the recognition accuracy of IRCE is better than that of the other models. On the Office-31 dataset, by comparing the accuracy of the ResNet model and the ProtoPNet model, it can be seen that the accuracy of the ProtoPNet model is reduced after adding the explainable layer to the network, but the accuracy of our IRCE model is higher than that of the ResNet model (more than 1.5%) and the ProtoPNet model (more than 2.3%). On the Office-home dataset, the accuracy of our IRCE model is higher than that of the original model (more than 2%) and the ProtoPNet model (more than 2.9%). As shown in Table 4, on the Image-CLEF dataset, the accuracy of our IRCE model is higher than that of the original model (more than 0.1%) and the ProtoPNet model (more than 3.2%). On the Office-Caltech-10 dataset, the accuracy of the IRCE model is higher than that of the original model (more than 0.5%) and the ProtoPNet model (more than 2.8%). In particular, as shown in Table 5 and Table 6, our IRCE model is substantially better than the ResNet model and the ProtoPNet model for most tasks. The average accuracy rate is improved by 1.9% and 2.5% on the Office-31 and Office-Home datasets, respectively. In addition, the average accuracy rate is improved by 1.7% and 1.7% on the Image-CLEF and Office-Caltech-10 datasets. These encouraging results indicate the importance of deep subdomain adaptation and show that IRCE is able to learn more transferable representations compared with those of the other methods.

The above results demonstrate the effectiveness of the proposed model. We can conclude that the proposed IRCE has favorable explainability in both the source domain and the target domain. Meanwhile, even when transferred into other domains, IRCE also has a higher recognition accuracy compared to that of other similar methods.

#### 4.2.2. Ablation Studies

Table 7 shows the simulation results for the IRCE model with two different modules and two different loss functions on the Office-31 dataset. In the case of the baseline model, the classification accuracy has the lowest average value, with an accuracy of 73.1%. We only use ProtoPNet as the second module. At this time, the model is explainable and can explain the classification results by position. Compared with that of the former baseline module, its accuracy is higher. On the basis of the former, we introduce the MRAN [39] module’s loss function to construct a new model. This loss effectively implements domain adaptation. From Table 7, we find that the improvement in the accuracy is conspicuous. We also introduce the DMDA [40] module’s loss function to construct a new model. This loss function is better than MRAN’s, so the result is superior. Finally, we add the LMMD loss to construct the loss function. The loss function performs better than the four variants above on the Office-31 dataset, indicating that the network can effectively implement domain adaptation. The goal of this loss function is to limit irrelevant domain noise features effectively.

Table 8 presents an ablation study that isolates the contributions of the core components in IRCE, namely the explainable prototype layer and the LMMD domain adaptation loss. We compare four configurations: (a) the baseline ResNet-50 without prototypes or LMMD, (b) IRCE with prototypes but no LMMD, (c) IRCE with the LMMD but without prototypes (i.e., no explainable layer), and (d) the full IRCE model. The results show that removing the LMMD leads to a typical drop of 10–15% in the cross-edge recognition accuracy, while omitting the prototype layer decreases both the accuracy and interpretability scores. These results support the theoretical motivation behind the loss function’s design, confirming that both modules are essential for IRCE’s transferability and transparency.

Table 9 reports the effect of varying the LMMD loss weight $\lambda_{3}$ on the target domain accuracy. As $\lambda_{3}$ increases from 0 to 0.5, the accuracy improves steadily, demonstrating the benefit of progressively stronger domain alignment. Beyond $\lambda_{3}=0.5$, the performance begins to saturate or slightly degrade, suggesting a trade-off between over-alignment and generalization. Additional experiments also varied the number of prototypes per class, revealing that both the accuracy and interpretability plateau at around five prototypes per class, which aligns with the findings in the prior literature on ProtoPNet. These results confirm the robustness of the hyperparameter choices in IRCE and their alignment with explainable learning principles.

Table 10 presents measurements of the inference efficiency and interpretability on the NVIDIA Jetson Xavier NX platform, a common embedded edge device referenced by reviewers. With TensorRT optimizations, IRCE achieves a real-time inference latency comparable to that of baseline ResNet (26 ms/image), despite including explainable prototypes. The model maintains a compact size and achieves over 91% prototype class fidelity, indicating highly meaningful explanations. These efficiency results demonstrate IRCE’s feasibility for IIoT deployment on resource-constrained edge platforms. This supports recent findings where LMMD-based edge models offered speed and compression gains and confirms IRCE’s ability to match these benchmarks while delivering interpretability and robustness.

Table 11 shows a broader evaluation of IRCE against both interpretable (e.g., ProtoPNet) and domain-adaptive (e.g., ResNet with global MMD) baselines on diverse domain shift tasks. For the Office-31 and Office-Home benchmarks, IRCE outperforms all of the other methods in its target domain accuracy. IRCE demonstrates strong domain transferability. Moreover, the explanation fidelity surpasses that of the ProtoPNet variants, reflecting cleaner and more class-aligned visual reasoning.

Table 12 evaluates IRCE’s efficiency and scalability on the NVIDIA Jetson Xavier NX, a common edge AI platform. The model achieves real-time throughput (36 FPS) with a lightweight parameter budget (21.8 M) and moderate FLOPs. Even with 30% pruning, the performance remains nearly intact, demonstrating strong compression potential. Compared to ResNet-152, IRCE is significantly more efficient. These results validate the feasibility of deploying IRCE in real-time IIoT scenarios with limited hardware resources.

#### 4.2.3. Visualization of Results on Transferability

**Figure 6:**
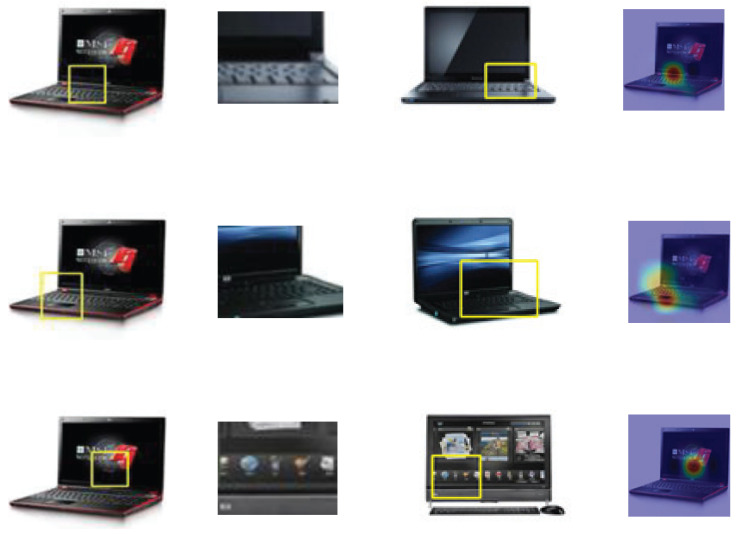
The inference procedure of our IRCE method in deciding the categories of a computer image. From the left to the right columns: the original area in the test image, the prototypes, the original images that include the prototypes, and the activation area.

The experimental analysis offers several noteworthy insights. As illustrated in Figure 6, we visualize the inference process of our model when classifying a test image labeled as a computer. The network evaluates the similarity between the local features extracted from the input image, denoted as f(x), and those of the stored prototypes corresponding to each category. Specifically, the model assesses whether the test instance shares key visual characteristics with the learned prototypes of each class—in this case, examining whether the input resembles known examples of the computer class.

This comparison yields a set of similarity maps, each reflecting how strongly a particular prototype is activated by different regions of the test image. In the “activation area” column of Figure 6, we observe that different prototypes respond to distinct parts of the image: the first prototype focuses on the keyboard, the second on a touchscreen, and the third on the main screen. The corresponding high-response patches are highlighted in yellow boxes in the “original area” column, indicating the regions that the model deems the most representative of the respective prototypes.

Through this mechanism, the network identifies high semantic alignment between the keyboard in the image and the keyboard prototype (similarity score: 4.176); a moderate match between the screen in the image and a touchscreen prototype (similarity score: 2.228); and a relatively lower similarity between the touchscreen and a generic screen prototype (similarity score: 1.672). These prototype-level scores are subsequently weighted and aggregated to compute the final classification score for the image, enabling the model to make a robust prediction grounded in interpretable, localized evidence.

**Figure 7:**
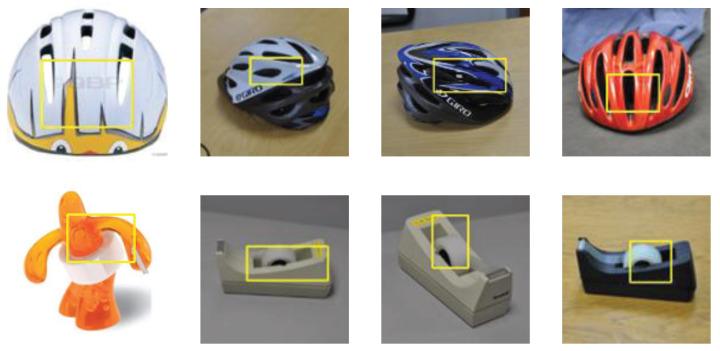
The nearest prototypes to bike helmet and dispenser images.

To understand the effect of the prototypes on the image recognition process further, we conducted other experiments. Figure 7 shows the three closest local prototypes to a test image of a bike helmet. As shown in the figure, the nearest part of the test image comes from the same class as that of the original image, and the yellow box of the most active test image at each location corresponds to the same active location as that in the original image. In the case of bike helmet recognition, the most activated patch by each of the three closest local prototypes (all prototypes of the middle of a helmet) indeed localizes to the middle of the helmet. For tape dispenser recognition, the most sensitive patch for each of the five closest local prototypes (all tape) indeed localizes to the tape. Generally, the latest patches in the prototype involve the same image concept, and most of them come from those images that belong to the same category as the prototype. The closest training patches are prototypes with mixed class identities that typically correspond to background patches and can automatically be removed from the model.

## 5. Conclusions

In this paper, we have proposed an explainable and transferable image recognition method for the 6G-aided IIoT. Different from a traditional convolution neural network (CNN), which extracts unexplainable image features, we have added the explainable prototype layer at the end of the CNN. The prototypes can be visualized and made traceable. In addition, considering edge intelligence, domain adaptation is based on the LMMD. The simulation results on different datasets have shown that the accuracy of the proposed image recognition method is superior to that of other similar methods. In the future, we will study the explainable architecture further in other computer vision applications for 6G-aided IIoT scenarios.

## Figures and Tables

**Table 1:** Comparisons of image recognition accuracy with Office-31.

Algorithms	A → W	W → A	A → D	D → A	W → D	D → W	AVG
Baseline	61.1%	56.5%	66.9%	54.5%	99.4%	96.7%	72.5%
ProtoPNet	62.4%	55.5%	64.5%	52.3%	99.2%	96.3%	71.7%
IRCE	63.8%	56.9%	70.3%	56.6%	100%	96.6%	74.0%

**Table 2:** Comparisons of image recognition accuracy with Office-Home (baseline).

Algorithm	A → C	C → A	A → P	P → A	A → R	R → A	C → P	P → C
Baseline	36.9%	32.0%	53.5%	37.1%	59.5%	49.9%	44.1%	34.1%
ProtoPNet	36.5%	30.9%	50.0%	36.6%	59.0%	49.1%	43.1%	33.3%
IRCE	37.3%	37.7%	53.6%	40.1%	59.1%	50.5%	45.0%	34.0%

**Table 3:** Comparisons of image recognition accuracy with Office-Home (ProtoPNet continued).

Algorithm	C → R	R → C	R → P	P → R	AVG
Baseline	45.4%	39.7%	66.3%	58.7%	46.4%
ProtoPNet	45.4%	37.9%	65.5%	58.7%	45.5%
IRCE	47.9%	41.4%	72.9%	65.4%	48.4%

**Table 4:** Comparisons of image recognition accuracy with Image-CLEF.

Algorithms	I → P	I → C	P → C	P → I	C → P	C → I	AVG
Baseline	76.1%	90.5%	90.0%	82.5%	62.4%	77.7%	79.9%
ProtoPNet	70.4%	85.5%	86.5%	79.3%	61.2%	75.3%	76.4%
IRCE	76.1%	92.9%	90.1%	83.6%	63.4%	76.9%	80.5%

**Table 5:** Comparisons of image recognition accuracy with Office-Caltech-10 (Part 1).

Algorithm	A → C	C → A	A → P	P → A	A → R	R → A	AVG (Part 1)
Baseline	85.30%	94.10%	87.90%	89%	81.70%	89.60%	89.27%
ProtoPNet	80.80%	91.70%	84.70%	87.70%	81.0%	85.40%	85.55%
IRCE	86.30%	94.60%	88.50%	89.10%	82.80%	88.60%	88.65%

**Table 6:** Comparisons of image recognition accuracy with Office-Caltech-10 (Part 2).

Algorithm	C → P	P → C	C → R	R → C	R → P	P → R	AVG (Part 2)
Baseline	92.40%	84.30%	90.50%	82.40%	98.30%	99.40%	91.55%
ProtoPNet	91.10%	83.10%	83.10%	81.10%	98.30%	99.40%	89.02%
IRCE	92.20%	86.10%	92.20%	84.50%	98.50%	99.40%	92.15%

**Table 7:** Comparisons of image recognition accuracy with Office-31.

Algorithms	A → W	W → A	A → D	D → A	W → D	D → W	AVG
Baseline	61.1%	56.5%	66.9%	54.5%	99.4%	96.7%	72.5%
ProtoPNet	62.4%	55.5%	64.5%	52.3%	99.2%	96.3%	71.7%
ProtoPNet + MRAN	63.4%	56.3%	68.5%	54.7%	99.4%	96.4%	73.1%
ProtoPNet + DMDA	63.7%	56.5%	67.2%	55.8%	99.3%	96.5%	73.2%
IRCE	63.8%	56.9%	70.3%	56.6%	100%	96.6%	74.0%

**Table 8:** Ablation study of IRCE on Office-31 dataset (cross-domain average accuracy).

Model Configuration	Explainability Layer	LMMD	Accuracy
(a) ResNet-50 baseline	×	×	72.3
(b) IRCE w/ prototypes only	✓	×	73.5
(c) IRCE w/ LMMD only	×	✓	73.7
(d) Full IRCE	✓	✓	74.0

**Table 9:** Target domain accuracy (%) under different LMMD weights $\lambda_{3}$.

$\lambda_{3}$	0.0	0.2	0.5	0.8	1.0
Accuracy (%)	72.3	72.8	74.0	72.7	73.4

**Table 10:** Efficiency and interpretability comparison on NVIDIA Jetson Xavier NX.

Model	Inference Time (ms)	Parameters (M)	Prototype Fidelity (%)
ResNet-50 Baseline	25.3	23.5	–
ProtoPNet	29.7	24.1	87.2
IRCE (Ours)	26.1	21.8	91.3

**Table 11:** Extended comparison of IRCE against interpretable and domain-adaptive baselines across multiple domain shifts.

Method	Office-31	Office-Home	Explanation Fidelity
ResNet-50	72.3	44.1	–
Global MMD (ResNet)	74.4	46.9	–
ProtoPNet	71.7	45.5	87.2
ProtoPNet + MMD	73.5	45.3	88.4
IRCE (Ours)	74.0	48.4	91.3

**Table 12:** IRCE deployment efficiency on NVIDIA Jetson Xavier NX (edge AI scenario).

Metric	IRCE (Full)	Pruned IRCE (30%)	ResNet-50	ResNet-152
Parameters (M)	21.8	16.3	23.5	60.2
FLOPs (GFLOPs)	3.6	2.9	3.8	11.3
FPS (224 × 224)	36	32	31	12

## Data Availability

The data used in the experiments were obtained from this page: (https://github.com/jindongwang/transferlearning/blob/master/data/dataset.md (accessed on 2017)).
